# Awareness Regarding Human Papilloma Virus Among Health Professionals and Will to Accept Vaccination: A Systematic Review

**DOI:** 10.7759/cureus.30855

**Published:** 2022-10-29

**Authors:** Efthymia Thanasa, Anna Thanasa, Evangelos Kamaretsos, Ioannis Paraoulakis, Konstantina Balafa, Ektoras-Evangelos Gerokostas, Gerasimos Kontogeorgis, Nikoleta Koutalia, Dimitra Stamouli, Vasiliki Grapsidi, Efthymia Alexopoulou, Georgia Ntella, Elena Sfondyli, Ioannis Thanasas

**Affiliations:** 1 Medicine, Aristotle University of Thessaloniki, Thessaloniki, GRC; 2 Obstetrics and Gynecology, General Hospital of Trikala, Trikala, GRC; 3 Obstetrics and Gynecology, General Hospial of Trikala, Trikala, GRC; 4 Obstetrics and Gynecology, General Hospital in Trikala, Trikala, GRC; 5 Obsretrics and Gynecology, General Hospital of Trikala, Trikala, GRC

**Keywords:** health professionals, acceptance of hpv vaccination, awareness, knowledge, hpv vaccines, hpv

## Abstract

Human Papilloma Virus (HPV) infection is the most common sexually transmitted disease and the leading cause of cervical cancer. The undeniable causal link between HPV and cervical cancer led to the creation of HPV prophylactic vaccines. Health professionals are key in counseling parents about their children's immunization, as they are considered valid and reliable sources of information. The systematic review aimed to determine doctors' and nurses' knowledge of HPV, their awareness of the vaccine, and their willingness to accept vaccination. Systematic studies were conducted from 2015 to January 2022 in Medline/PubMed and Google Scholar online databases. The systematic review included 10 good-quality cross-sectional studies and a total of 6700 participants who were administered self-administered questionnaires or personal interviews. From the analysis of most of the studies, it is demonstrated that health professionals have a satisfactory level of knowledge about HPV infection and its effects on human health, even if their knowledge gap in essential details regarding the virus and HPV vaccination is apparent. It was found that various factors regarding health professionals, such as their specialty, gender, working environment, weekly working hours, and the interval since their last HPV training, contribute to forming their knowledge level about HPV and vaccination. In addition, most studies show that most healthcare professionals knew about the existence of HPV vaccines but did not know many details about how their work and their potential benefits. In conclusion, the provision of counseling by health professionals is currently estimated to be the strongest predictor of target group compliance with the HPV vaccine. Consequently, it is essentially considered to investigate the HPV-related knowledge level among health workers and to intensively reeducate them regarding the HPV infection risks and the necessity of HPV vaccination to improve their awareness and strengthen their attitude in favor of vaccination against cervical cancer.

## Introduction and background

Human Papilloma Virus (HPV) is a small DNA virus that interacts with squamous epithelium. HPV infection is the most common heterosexual and homosexual sexually transmitted disease worldwide [1,2]. Although most HPV infections are asymptomatic, transient, and spontaneously resolved within about two years after the exposure, it is estimated that 10%-20% of infections persist and may lead to various forms of invasive cancer. The global prevalence of high-risk HPV infection is 10.4%, [3] with sexual activity estimated to influence the risk of genital infection [4] significantly. In developing countries, the risk of infection with high-risk HPV strains is estimated to reach 36.5% [5].

More than 170 different HPV types have been identified that infect stratified epithelium [6]. Depending on the risk of oncogenesis, HPV types are divided into high and low risk. HPV 16 and 18 are the most common genotypes in cervical cancer and account for approximately 70% of cases. In contrast, low-risk HPVs, the most frequent of which are HPV 6 and 11, are detected in acute warts and low-grade cervical intraepithelial lesions (LSIL) but do not cause cancer [7].

Persistent viral infection with high-risk HPV genotypes causes almost all cervical cancers (99.7%): 60% of vulvar cancers, 70% of vaginal cancers, and 93% of anal cancers [8-10]. HPV accounts for more than 5% of cancers worldwide, including all cervical cancers and an alarmingly increasing proportion of oropharyngeal cancers [11]. Cervical cancer is the second most common cancer in women, with more than 250.000 deaths per year worldwide [12].

Establishing a causal relationship between HPV and cervical cancer led the scientific community to decide to create HPV prophylactic vaccines. Today, three vaccines are available: the bivalent vaccine, which protects against types 16 and 18; the quadrivalent against types 6, 11, 16, and 18 and the nine-valent HPV vaccine, which protects against types 6, 11, 16, 18, 31, 33, 45, 52 and 58. Despite the wide availability of prophylactic HPV vaccines over the past 15 years, vaccination coverage remains low [13]. In low- and middle-income countries, where the incidence of cervical cancer is high, it is estimated that by achieving vaccination coverage of 70%, more than 4 million women's deaths could be avoided in the next decade [14]. Similarly, the new recommendations for routine vaccination in the male population are estimated to significantly reduce oncogenesis in both males and females and help achieve the desired immunity [15]. The purpose of this critical literature review was to record worldwide the knowledge of health professionals regarding HPV and its infection, as well as to determine their awareness of the HPV vaccine and willingness to accept vaccination against cervical cancer.

## Review

Methodology

The method used with the research hypothesis of our study is the systematic review of the literature. Our study aims to determine health professionals' knowledge about HPV, awareness of the vaccine against the virus, and willingness to accept HPV vaccination. Studies conducted up to December 2021 were included. The systematic bibliography review aims to identify, assess, and select well-designed studies relevant to our research hypothesis according to predetermined and clear scientific criteria. After defining the research question and searching for the relevant studies in the international literature, the inclusion and exclusion criteria of the studies, the searching process flowchart and selected studies, the summary table, and the evaluation of the quality of the selected studies are in each case steps of decisive and prominent importance for the successful realization of the systematic literature review.

Description of Search Strategy

The international literature search was carried out in the electronic database Medline/PubMed and Google Scholar with keywords, as shown in Table 1.

**Table 1:** Keywords for the systematic search for studies exploring healthcare professionals' knowledge of HPV and willingness to accept HPV vaccination in the Medline/PubMed and Google Scholar databases.

HPV OR Human Papilloma Virus OR Human Papilloma Viral Infection Epidemiology Study OR Descriptive Study
Knowledge OR Awareness OR Learning Ability OR Behaviour OR Attitude Adolescent OR Adolescence
Vaccine OR Vaccination OR HPV Vaccination Programme OR Implementation of Vaccination Programme OR Attitude Toward to Vaccine OR Immunization OR Coverage

The literature search was also carried out through additional records relevant to the subject under investigation and resulted from searching other data sources, such as communication with specialized health scientists, conference proceedings, and doctoral thesis.

Study Inclusion and Exclusion Criteria

The search was based on the following study inclusion and exclusion criteria:

Language of publication: Research published in the English language.

Date of publication: Research published after 2015.

Study content: Surveys analyzing the relationship between recording health professionals' knowledge of HPV and acceptance of HPV vaccination.

Type of Study: Contemporary studies, cohort studies, observational studies, and quantitative studies published in scientific journals and conducted in different countries can be included. Only studies of which the full article is accessible will be included, considering that the study's design has recorded the available literature without altering the final non-results.

Study sample: The sample size of the included studies should be >-50 to consist of male and female health professionals.

Reporting: Participants in the included studies should answer the questionnaires or interviews by themselves; in studies where others answered the questionnaires, the study participants will be excluded.

Outcome: Studies demonstrating the relationship (or lack thereof) between HPV knowledge and awareness and acceptance of vaccination will be included in the review. Ideally, studies that identify the influencing factors and the best interventions to increase awareness of the HPV vaccine will also be included.

Studies from the most authoritative medical science journals, all peer-reviewed, were included.

Flow Chart

**Figure 1:**
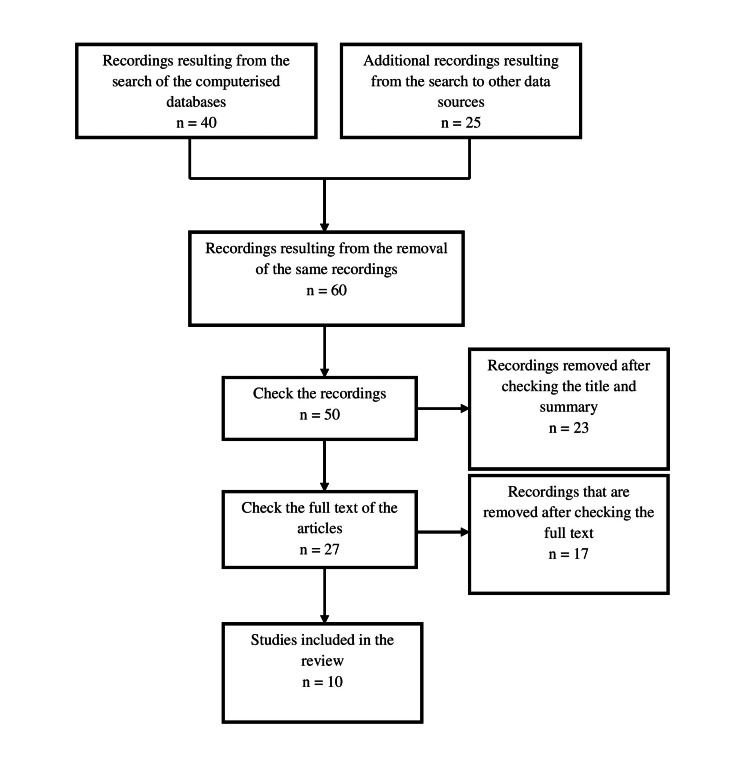
Flow chart of the search and selection process of the studies included in the systematic review.

The visualization of the results of the strategic research was done with the flowchart (Figure 1), in which the process of searching and selecting the studies is briefly presented [16].

Selection and Presentation of Studies

Table 2 presents the articles and studies found, according to the relevant topic search, as described in the methodology.

**Table 2:** Aggregate table of studies selected to be included in the systematic literature review.

First author's name	Year	Country	Participants	Type of Study	Identifier (Research tool)	Outcome	Results
Chawla et al.	2016	India	590	cross-sectional study	Questionnaire self-administered validated	Only 47% of respondents recommended young women get vaccinated against HPV. 81% were aware of the existence of vaccines to prevent cervical cancer. Wide differences were observed between the different regions with health professionals, with urban areas showing higher rates (88.3%) of knowledge about the vaccine compared to rural areas (64%). Although 86% of gynecologists were aware of the names of HPV vaccines available in the market, only 27% of paramedical staff had this knowledge. There was a significant difference between respondents from the public and private sectors in terms of their awareness of HPV vaccines. Lack of awareness of the principal cause, risk factors and symptoms of cervical cancer and HPV vaccination was found statistically significant (p<0.05) among respondents from the paramedical staff category.	The findings reinforce the continued medical education of healthcare providers, particularly those from the public sector on HPV vaccination for cervical cancer prevention. Public education is also essential for a successful HPV vaccination program in the country
McSherry et al.	2018	Ireland	697	cross-sectional study	A questionnaire including questions related to HPV infection and vaccination	For HPV infection, general practitioners and nurses answered a median of nine and seven questions correctly, respectively (p <0.001). Significantly associated with the low knowledge about HPV infection were: being a nurse / male general practitioner, working fewer hours/week, working in the private sector, and not engaging in gynecological issues. For the HPV vaccine, both GPs and nurses answered a mean of six questions correctly (p = 0.248). Significantly associated with the low knowledge of HPV vaccination were: the male sex of nurses, and general practitioners, working more years in general medicine, fewer hours per week, in a small practice or in a practice that does not specialize in women's health. Six questions concerning HPV infection and seven questions concerning HPV vaccination were not answered correctly by >⅓ of healthcare professionals	There are significant limitations to the knowledge of HPV infection and vaccination among health professionals. By identifying factors related to insufficient knowledge and areas of particular uncertainty, these results can inform the development of vocational training initiatives
Sherman et al.	2018	New Zealand	230	cross-sectional study	Questionnaire exploring 4 general areas: demographics and level of experience, knowledge about HPV and vaccination, attitudes towards the HPV vaccine, and self-perceived adequacy of knowledge about HPV	The average score on the general HPV knowledge questions was 13.2/15, with only 25.2% of respondents scoring 100%. The average score on the knowledge questions for the diagnosis of HPV and the test of cure was 7.4/10, with only 9.1% being rated 100%. The average score on the knowledge questions about the HPV vaccine was 6/7 and 44.3% scored 100%. Only 63.7% of respondents agreed that they were adequately informed about HPV, although 73.3% agreed that they could confidently answer HPV-related questions posed by patients. Multifactorial analyses revealed that knowledge in each domain predicted confidence in responding to patient questions. In addition, the number of years of training provided both knowledges of HPV and knowledge of diagnostic and treatment procedures.	Although the overall level of knowledge was adequate, there were significant knowledge gaps, particularly regarding the role of the HPV test in the New Zealand National Cervical Screening Program. More training is needed to ensure that misinformation and stigma do not inadvertently arise from interactions between health professionals and the public.
Pereira et al.	2019	Brazil	196	cross-sectional study	Questionnaire of 31 questions about HPV and the clinical significance of the vaccine was completed in the form of interviews	Doctors had increased knowledge only about the statement "cervical cancer is one of the main causes of cancer in women", with a ratio of 0.88 (0.80; 0.97) and p <0.001. Regarding the clinical significance of the HPV vaccine, a low percentage of correct answers was obtained for all questions, and no significant differences were found between the occupational groups.	Acceptance and knowledge of HPV and the vaccine were similar among healthcare professionals, with knowledge gaps in questions about the relationship between smoking and cervical cancer and specific clinical knowledge
Sherman et al.	2020	Great Britain	634	cross-sectional study	A questionnaire designed to assess general awareness of HPV. Deliberation of knowledge about the diagnosis and treatment of HPV and knowledge about the HPV vaccine. It also examined the attitudes to the HPV vaccine and the self-perceived sufficiency of knowledge.	Participants generally had a good understanding of HPV and vaccination, but there were gaps in detailed knowledge of HPV test procedures. There have been some gaps in knowledge about the effects of HPV on men's health. Years since HPV training was associated with diagnosis, treatment, and vaccine knowledge scores. Furthermore, nurses and doctors in colposcopy clinics were much more likely to have higher knowledge across all domains than in other roles. Overall, 76.2% of participants felt adequately informed about HPV and 35.6% suggested improvements in education, many of whom requested additional frequency and topics.	The results denote that additional training is needed to ensure that health professionals are equipped to deal with the changing landscape of HPV screening and vaccination in the UK
Trucchi et al.	2020	Italy	1410	cross-sectional study	Anonymous questionnaires addressed to health workers	The overall mean knowledge and attitude score was 69.2% (25–75, p = 61.5–84.6) and 5 (25–75, p = 4–5), respectively. Both knowledge and attitudes differ statistically significantly between physicians and other health professionals. The median propensity score before and after the educational intervention was stable and high, at 10 (25–75, p = 9–10). The predictors of statistically significant high knowledge scores are being a doctor, general practitioner, or pediatrician, attending courses/congresses, and consulting the technical characteristics of the product and the scientific literature to obtain information about the HPV vaccine. Being a physician and consulting of scientific literature to obtain information about the HPV vaccine was also found as prognostic factors statistically significantly different attitude scores among the participants in the study.	Although healthcare professionals have generally shown a positive attitude toward the relevance of HPV health burden and prevention tools, the proven knowledge was largely suboptimal, especially that shown by healthcare professionals. The results make it possible to identify knowledge gaps and, consequently, to improve counseling to HPV vaccine targets.
Xenaki et al.	2020	Greece	750	cross-sectional study	Questionnaire of 31 questions about the knowledge, attitude and behavior of educators and health professionals regarding the prevention of HPV	Of the total participants, 26.4% had excellent knowledge, 44.7% had good, 23.6% had moderate, 4.7% had poor knowledge and 4.7% had a very bad level of overall knowledge. Specifically, physicians of different specialties had 80.3% and educators had 65.3% excellent level of knowledge, respectively. Also, the participants had a high degree of awareness about the prevention and observance of the annual Papanikolaou smear test (65.8%) and the gynecological examination. Despite the positive response (94.1%) regarding the vaccination of boys and girls, as well as the information about the HPV vaccination, many participants have not vaccinated themselves (74.8%), nor their children (19.7%).	The present study showed a good level of knowledge for the prevention of HPV regardless of occupation and is characterized by a high degree of awareness of the usefulness of prevention in the observance of the annual Papanikolaou smear test and gynecological examination. The study shows the need for more information workshops for healthcare professionals because doctors and midwives had high levels of knowledge, but not excellent as expected and required due to a health-related profession.
Yu et al.	2020	China	1249	cross-sectional study	A questionnaire of 41 questions was designed based on questionnaires that had been published in the past. The main content of the interviews included: (1) Characteristics, (2) Knowledge about HPV and vaccination, (3) Reasons for willingness or reluctance to vaccinate and acceptable costs, (4) Factors related to willingness to promote vaccination	The level of knowledge about HPV was found to be low, but more than 80% of participants would like to receive the HPV vaccine. The medical staff was more willing to recommend the HPV vaccine than medical students (OR = 4,696, CI: 2,698-8,175), which may be related to work experience and acceptance of the vaccine price, but not to the level of knowledge.	The lack of knowledge is not a major factor influencing the willingness to vaccinate, but the level of knowledge about HPV needs to be increased. When the level of knowledge is high, education and per capita household income they are not significant factors influencing the willingness to vaccinate. The price of vaccines significantly affects the willingness to vaccinate. Incorporating the HPV vaccine into the national vaccination program could help alleviate public concerns about the vaccine to change the current situation.
Ebu et al.	2021	Ghana	318	cross-sectional study	A questionnaire with data adapted from previous studies, to determine the knowledge of cervical cancer and cervical cancer screening behavior of nurses and midwives in Ghana	The results showed that 41.5% of the participants had high levels of knowledge about the risk factors for cervical cancer and 17.6% of the participants had received at least one dose of the HPV vaccine. The reasons for receiving the HPV vaccination included advice from a colleague (12.9%) and a perceived threat of cervical cancer (11.7%). Of the 262 respondents who had not been vaccinated, 24.45% strongly agreed and 28% agreed with the statement that there was limited information about HPV vaccination. There were also statistically significant correlations between age (p=0.001), marital status (p=0.005), completed level of education (p=0.001), and duration of working at the hospital (p=0.038).	This study showed gaps in knowledge about risk factors for cervical cancer and attitudes toward HPV vaccination, indicating the need for targeted measures to improve knowledge and attitudes. Also, measures to increase the acceptance of HPV vaccination among nurses and midwives should take into account their socio-demographic characteristics.
Chowdhury et al.	2022	Bangladesh	626	cross-sectional study	A validated and structured questionnaire consisting of four extensive areas: socio-demographic characteristics, knowledge about HPV, attitudes, and practices regarding vaccination	The standards of knowledge and practice demonstrated very poor outcomes, where 43.29% of the respondents showed good knowledge and only 11.82% conveyed good practices. However, attitudes towards HPV vaccination were revealed high (75.88%). Female participants showed more positive attitudes than men.	Health professionals play a vital role in increasing public knowledge about HPV and raising awareness about HPV vaccination programs. The provision of medical training for HPV should be a priority and current training techniques re-evaluated. Thus, by adhering to this strategy an improvement in the national vaccination policy can be expected.

Evaluation of the Quality of the Studies

The evaluation of the studies, as shown in table 3, was done with the quality assessment scale for cross-sectional studies, as defined by the "Newcastle - Ottawa Quality Assessment Scale Case-Control Studies/Cohort Studies".

**Table 3:** Evaluation table for the quality of studies based on the Newcastle – Ottawa scale for cross-sectional studies.

STUDY	SELECTION				COMPARABILITY		EXPOSITION			TOTAL	QUALITY
Chawla et al, 2016	*	*	*	*	*	*	*	*	*	9	Low probability of bias
McSherry et al, 2018	*	*	*		*	*	*	*		7	Low probability of bias
Sherman et al, 2018	*	*		*	*	*	*		*	7	Low probability of bias
Pereira et al, 2019	*	*	*	*	*		*	*	*	8	Low probability of bias
Sherman et al, 2020	*	*		*	*	*	*		*	7	Low probability of bias
Trucchi et al, 2020	*	*	*		*		*	*		6	Moderate probability of bias
Xenaki et al, 2020	*	*	*	*	*	*	*	*	*	9	Low probability of bias
Yu et al, 2020	*	*	*		*	*	*		*	7	Low probability of bias
Ebu et al, 2021	*	*	*		*		*	*		6	Moderate probability of bias
Chowdhury et al, 2022	*	*		*	*	*		*	*	7	Low probability of bias

This scale assesses eight items and uses a star system with a maximum of 9 stars to evaluate non-randomized studies on three criteria: selection of patients or study groups (4 stars), comparability of study groups (2 stars), and its finding on the exposure or outcome of interest for case-control or cohort studies, respectively (3 stars). Studies that scored six stars or more are considered good quality. The above rating scale provides an easy and reliable way to assess the clarity, completeness, and quality of non-randomized studies to be used in a systematic review [17].

Results

Of the 60 studies that emerged from the systematic online search using the keywords, individually or in combination, as shown in table 1, 10 were selected for inclusion in this systematic review. Of the ten included studies assessed using the Newcastle-Ottawa rating scale (Table 3), eight were graded 7- 9 and classified as studies with low potential for bias. In comparison, 2 of the 10 studies were graded six and classified as the medium probability of bias studies. All studies were considered good quality and eligible for inclusion in the critical systematic review. In total, the studies in the systematic review included 6700 participants who were health professionals in different capacities. Also, all ten studies examined the level of knowledge about HPV and the willingness to accept vaccination of healthcare professionals in different countries of the world. The countries in which the studies included in the systematic review were conducted were high, middle, and low-standard-of-living countries, thus constituting a representative sample of health professionals in different health systems worldwide. All the included studies were cross-sectional studies that used self-administered questionnaires or personal interviews in the participants' language, so there was no limitation of language comprehension.

In most studies, it appears that health professionals worldwide, despite showing severe knowledge gaps in essential details regarding the virus and HPV vaccination, had a satisfactory knowledge level about HPV infection and its effects on women's and men's health. Also, most studies show that most healthcare professionals worldwide were aware of the existence of the HPV vaccine but did not know many details about how the vaccines work and the potential benefits associated with the HPV vaccination.

An important result of the present research study was that health professionals, especially those in low-income countries, are unwilling to recommend the HPV vaccine to young women and girls. Nevertheless, it is found that after completing some educational processes - interventions during the study, health professionals seem to change their attitude and are more willing to recommend the vaccine to their patients. This fact indicates the need for regular re-education of health professionals regarding the risks of HPV infection and the necessity of vaccination against cervical cancer.

Discussion

Several studies, mainly from developed countries, have shown that the level of knowledge about HPV, vaccines, and acceptance of prophylactic HPV vaccines among healthcare providers varies from low to high. Health professionals in both developed and developing countries play an essential role in immunization programs, especially those related to childhood diseases, as they are used to administer prophylactic HPV vaccines and contribute significantly to the health education of the general population. It is now estimated that the success of an immunization program against cervical cancer is highly dependent on health professionals' knowledge, attitude, and behavior towards HPV, the infection caused by the virus, and prophylactic HPV vaccines.

From the analysis of the results of the present study, it appears that worldwide the knowledge of health professionals about HPV is at a reasonably high level. It is estimated that 60%-80% of participants demonstrate a high to very high-level knowledge of HPV infection. Nevertheless, various factors may influence the level of knowledge of health professionals. In half of the studies included in the systematic review, it appears that the level of awareness of a high risk of oncogenic HPV types was observed to be significantly differentiated between health professionals of different specialties [18-22]. More specifically, general practitioners, gynecologists, pediatricians, and workers in obstetric nursing units (midwives, nurses) showed a higher awareness of high-risk HPV genotypes than the rest of the paramedical staff, whose relative rates of knowledge and awareness were too low.

Also significant were the differences observed in the level of knowledge about HPV and prophylactic vaccination between health professionals working in regional and urban health centers. Health professionals in urban centers presented a higher level of knowledge than their colleagues working in health centers in rural areas uniformly across all income countries [19,22]. In addition, McSherry et al. found that the male gender, particularly among nurses and general practitioners, and working fewer hours per week or working in smaller health centers were additional factors contributing to low levels of knowledge about HPV and vaccination [22]. Similar conclusions of the study by Sherman et al. showed that time since the last professional training on HPV was associated with gaps in knowledge regarding diagnosis, treatment, and HPV vaccines [18]. In 2020 Trucchi et al. confirmed the previous conclusions and also showed that health professionals who regularly attend educational programs-seminars on the technical characteristics of vaccines or had easier access to information about HPV and vaccines had a higher level of knowledge about HPV infection, the effects it can have on human health and the potential benefits of HPV vaccination, regardless of their specialty [21].

The present study also investigated participants' attitudes toward HPV vaccination and their willingness to be vaccinated or to recommend the vaccine to others. In most studies, it appears that although most participants had a moderate to a high level of knowledge about HPV, the knowledge regarding the characteristics and pharmacological properties was relatively low. This resulted in participants needing help to commit to participating in the vaccination program and recommending the vaccine to the population. More specifically, in 2016, Chawla et al. showed that only 47% of participants would recommend the HPV vaccination to young women, even though 81% were aware of vaccines to prevent cervical cancer [19]. Later in 2019, Pereira et al. concluded that the level of knowledge regarding the clinical importance of the HPV vaccine was found to be low, and no significant differences were found between the professional groups surveyed [23].

In contrast, a recent study showed that the medical staff was more willing to recommend the HPV vaccine than health professional students. This may be related to greater work experience and easier acceptance of the cost of the vaccine by healthcare professionals rather than the level of knowledge about HPV and vaccination. However, a high level of knowledge appears to positively influence willingness to be vaccinated, independent of education and per capita family income [24].

Also, Ebu et al., analyzing the reasons for a negative attitude toward vaccination, found that the largest percentage of unvaccinated participants agreed with the statement that there was limited information about HPV vaccination. Also, in the same study, statistically significant correlations were found between age, marital status, level of education, and duration of work in a health facility with the acceptance of HPV vaccination [25]. In contrast, the study by Xenaki et al. found that despite participants' positive response to vaccinating boys and girls and a good level of knowledge about HPV vaccines, many participants did not vaccinate themselves or their children. This was probably attributed to the fact that they compensated for low self-willingness to vaccinate with a high degree of awareness about adherence to annual Pap smears and gynecological examinations [25]. Many studies have found that the lack of information among participants about the leading cause, risk factors, and symptoms of cervical cancer is the main reason for the low participation rate of health professionals, especially paramedical staff, in vaccination programs or recommendations of the HPV vaccine [19,25-27].

An important finding of the present study is that health professionals had relatively good self-awareness regarding the level of knowledge about HPV and vaccination.

Specifically, in Sherman et al. 2018 study, it was found that 63.7% of participants agreed that they were adequately informed about HPV, and 73.3% agreed that they could confidently answer patients' questions about HPV [20]. However, most studies have shown that when participants received some form of education during the study, they felt more confident about their knowledge of HPV and vaccination. In a later study in 2020, Sherman et al. found that 76.2% of participants felt adequately informed about HPV and that 35.6% made suggestions for improvements in education [18].

The results of the present investigation suggest that education about HPV, particularly the use of HPV testing in the screening program, is necessary to address some worrying gaps in healthcare professionals' knowledge levels. The need for healthcare professional education is further reinforced by the fact that over a third of participants in the studies included in the systematic review did not agree that they felt adequately informed about HPV. Some made suggestions for training the health professionals of the participants. A good suggestion made by UK nurses was online education [28]. Online education is believed to provide a low-cost way to communicate changes in HPV vaccination or screening programs and guidelines in a format that is easily accessible and requires relatively little time to implement.

Also, the important results of the present research include the fact that knowledge about HPV infection and transmission was worse among medical students compared to medical staff. It cannot be ignored that a significant proportion of medical staff and a greater proportion of medical students are unaware of basic information about how HPV is transmitted. This fact is a strong reminder of the lack of education about HPV in developing and developed countries. It is imperative to formulate educational programs to improve the knowledge level of the community and health professionals. A recent study from Sweden showed that 68% of patients prefer to receive preventive knowledge about HPV from doctors and health professionals, which may be a method to help increase awareness worldwide [29].

Study limitations

This review is subject to certain limitations. First, there are studies by research groups published in a language other than English, with the result that the published material used may have been underrepresented. It is also possible that some studies explore the knowledge of HPV among healthcare professionals but did not record the other recommended keywords and were not included in the studies under analysis, as they were considered not to meet all the criteria. Finally, the design followed in this review has recorded the available literature without altering the final results.

## Conclusions

Health professionals play a key role in providing advice to parents on their children's immunization, as they are considered valid and reliable sources of information. Counseling by healthcare professionals is currently estimated to be the strongest predictor of compliance of target groups with the HPV vaccine. Therefore, it is considered very important to regularly and systematically investigate the knowledge related to HPV among health workers to improve their awareness and strengthen their attitude toward cervical cancer vaccination.

Healthcare professionals must be prepared to support their role, recommend the vaccine, and address their patients' potential concerns about HPV. To this end, health professionals should have access to multiple sources of information, such as seminars, conferences, and scientific literature, which can make a significant contribution through general education to increasing the level of knowledge and awareness about HPV and vaccination. Regarding the improvement of education for HPV, it is pointed out that continuous online medical training in the context of cervical cancer screening has favorable effects, especially in increasing knowledge and enhancing the adoption of clinical guidelines.
